# Major protease inhibitor drug resistance mutations among patients with virological failure on second-line antiretroviral therapy in Zimbabwe: a retrospective cross-sectional study

**DOI:** 10.1186/s12985-026-03255-6

**Published:** 2026-07-10

**Authors:** Justin Mayini, Tendai Washaya, Justen Manasa, Edward Zumbika, Shungu Munyati, Ryman Shoko, Christine Dahlke, Vinie Kouamou

**Affiliations:** 1 Biomedical Research and Training Institute, 10 Seagrave Road, Avondale Harare, Avondale, Harare Zimbabwe; 2 National University of Science and Technology, Bulawayo, Zimbabwe; 3 Faculty of Medicine and Health Sciences, University of Zimbabwe, Harare, Zimbabwe; 4 School of Medicine and Health Sciences, University of Lusaka, Lusaka, Zambia; 5 Institute for Infection Research and Vaccine Development, Hamburg, Germany; 6 Laboratory of immunoregulation and infectious Diseases, NIAID, NIH Clinical Center, Bethesda, USA; 7 Infectious Diseases Research Laboratory, Harare, Zimbabwe

**Keywords:** HIV-1 Subtype C, Protease Inhibitors, Drug Resistance Mutations, Virological Failure, Zimbabwe

## Abstract

**Background:**

Virological failure (VF) on protease inhibitor (PI)-based second-line antiretroviral therapy (ART) remains an important driver of HIV drug resistance (HIVDR) in sub-Saharan Africa. Despite widespread adoption of dolutegravir (DTG)-based first-line ART, many people living with HIV (PLWH) remain on PI-based second-line regimens. Data on major PI drug resistance mutations (DRMs) in Zimbabwe remain limited.

**Methods:**

We conducted a retrospective cross-sectional study of archived plasma specimens from PLWH with VF (viral load (VL) ≥ 1,000 copies/mL) receiving PI-based second-line ART in Zimbabwe between January 2021 and June 2025. HIV-1 *protease* and *reverse transcriptase* regions were Sanger-sequenced, and resistance was interpreted using the Stanford HIV Drug Resistance Database. Factors associated with major PI DRMs were evaluated using multivariable logistic regression.

**Results:**

Among 297 participants, 153 (51.5%, 95% confidence interval [CI] 45.8–57.2%) harboured major PI DRMs. The most frequent mutations were M46I (22.6%), V82A (19.2%), and I54V (17.2%). NRTI- and NNRTI-associated DRMs were present in 70.4% and 79.5% of participants, respectively, while 47.8% exhibited triple-class resistance. Predicted susceptibility to darunavir/ritonavir remained high (78.8%), despite resistance to atazanavir/ritonavir (45.5%) and lopinavir/ritonavir (50.8%). In multivariable analysis, increasing age (adjusted odds ratio [aOR] 1.04, *P* < 0.001), higher VL (aOR 1.55, *P* = 0.011) and male sex (aOR 1.59, *P* = 0.044) were independently associated with major PI DRMs.

**Conclusions:**

Major PI DRMs and multiclass HIVDR were common among PLWH with VF on PI-based second-line ART. Routine VL monitoring and early resistance testing are needed to detect and guide second-line ART failure in Zimbabwe.

**Supplementary Information:**

The online version contains supplementary material available at https://doi.org/10.1186/s12985-026-03255-6.

## Background

Human immunodeficiency virus (HIV) infection remains a major global public health challenge despite substantial advances in antiretroviral therapy (ART) scale-up and reductions in HIV-related morbidity and mortality [1]. Sub-Saharan Africa (SSA) continues to bear the greatest burden of the epidemic and accounts for most people living with HIV (PLWH) receiving lifelong ART [2]. Although combination ART has markedly improved survival and quality of life, long-term treatment durability and the emergence of HIV drug resistance (HIVDR) remain ongoing challenges. In many low- and middle-income countries (LMICs), including SSA, ritonavir-boosted protease inhibitor (PI)-based regimens historically formed the backbone of second-line ART following failure of non-nucleoside reverse transcriptase inhibitor (NNRTI)-based first-line therapy, in line with earlier World Health Organisation (WHO) recommendations [3]. Although dolutegravir (DTG)-based regimens are now preferred first-line therapy [4], PI-based regimens remain important for individuals with prior treatment failure, complex treatment histories, or those requiring third-line ART. Preserving PI susceptibility therefore remains essential for maintaining effective long-term ART sequencing within the national ART programmes.

Virological failure (VF) on PI-based second-line ART remains a major clinical challenge. Ongoing viral replication under ART pressure may lead to the emergence of major PI drug resistance mutations (DRMs), reducing susceptibility to commonly used boosted PI regimens and increasing reliance on more costly third-line therapies such as darunavir/ritonavir (DRV/r)-based regimens [4]. Across SSA, the prevalence of major PI DRMs among PLWH failing second-line ART ranges from approximately 18–22% in multi-country cohorts [5–7] to nearly 50% among heavily treatment-experienced populations [8–11], depending on treatment duration, adherence, viral load (VL) monitoring practices, and access to genotypic resistance testing (GRT).

Zimbabwe has made good progress in HIV programme implementation and is among the first countries in SSA to achieve the UNAIDS 95–95–95 targets among adults [12, 13]. Recent estimates indicate that approximately 1.3 million PLWH are receiving ART nationally, with reported ART coverage and viral suppression rates of 97.0% and 95.0%, respectively [14, 15]. In line with WHO recommendations, Zimbabwe rapidly transitioned to DTG-based first-line ART and has subsequently reported high rates of virological suppression exceeding 90% among individuals receiving DTG-based regimens [16, 17]. Nevertheless, PI-based regimens remain important for PLWH with historical first-line NNRTI failure, complex treatment histories, or the need for regimen optimisation. Continued HIVDR surveillance within this sub-population therefore remains essential for guiding treatment policy and preserving long-term treatment effectiveness as national ART programmes mature in the DTG era [5].

Despite the continued importance of PI-based regimens, contemporary data on major PI DRMs in Zimbabwe remain limited. Previous studies among Zimbabwean PLWH receiving ART were often restricted by small sample sizes, limited geographic representation, or specific patient sub-populations [8, 18, 19]. Recent reports of multiclass and pan-resistant HIV-1 strains in Zimbabwe further underscore the growing threat posed by HIVDR within the national ART programmes [20]. This study therefore aimed to determine the prevalence of major PI DRMs, characterise resistance patterns, and identify demographic and clinical factors associated with major PI DRMs among PLWH experiencing VF on PI-based second-line ART in Zimbabwe.

## Methods

### Study design

This multicentre retrospective cross-sectional study included treatment-experienced PLWH with VF after ≥ 12 months on PI-based second-line ART in Zimbabwe between January 2021 and June 2025, using archived plasma specimens generated through the National HIVDR programme for GRT among individuals with suspected treatment failure. Consecutive eligible specimens received during the study period were included to minimise selection bias and better reflect programme-based second-line VF cases. This is a programme that supports routine GRT among PLWH with suspected treatment failure across public healthcare facilities to enable early detection of individuals with HIVDR. Plasma samples were collected from eight provincial and three central hospitals across the country and transported to the Biomedical Research and Training Institute (BRTI), Harare, using standardised national procedures for centralised laboratory processing, sequencing, and resistance interpretation. Demographic and clinical information were obtained from laboratory GRT request forms. The study aimed to determine the prevalence, patterns, and predictors of major PI DRMs among PLWH experiencing VF on PI-based second-line ART in Zimbabwe.

### Participants and eligibility criteria

Eligible participants were PLWH receiving second-line ART, defined as a ritonavir-boosted PI-based regimen, with confirmed VF. VF was defined as two consecutive VL measurements ≥ 1,000 copies/mL obtained at least six months apart, in accordance with national HIV treatment guidelines. ART regimen status, VL history, and eligibility for PI-based second-line ART were verified using laboratory GRT request forms and accompanying programme records. Participants were excluded if they were not receiving PI-based second-line ART, lacked VL data confirming VF, or had insufficient specimen volume or poor-quality specimens that precluded successful GRT. No age restriction was applied. Only one specimen per participant was included in the final analysis. No formal a priori sample size calculation was performed because this was a retrospective analysis of all available eligible archived specimens. The final analytic sample of 297 participants provided adequate precision for estimation of the primary prevalence outcome.

### Sample processing and laboratory procedures

Whole blood specimens (4 mL) were collected into EDTA tubes at the healthcare facilities and centrifuged within two hours of collection. Plasma was aliquoted and VL quantified using the Cepheid HIV-1 VL assay on the GeneXpert platform (Cepheid, CA, USA), with a lower limit of detection of 40 copies/mL. Specimens from participants meeting the national definition of VF (two consecutive VL measurements ≥ 1,000 copies/mL obtained at least six months apart) were transported frozen to BRTI for storage at − 80 °C prior to analysis. HIV-1 ribonucleic acid (RNA) was extracted from plasma samples using the NucliSens EasyMag system (bioMérieux, France), according to the manufacturer’s instructions. Reverse transcription and first-strand synthesis were performed using the Applied Biosystems SuperScript III One-Step RT-PCR Kit (Thermo Fisher Scientific, Waltham, MA, USA) on the Applied Biosystems MiniAmp Plus PCR thermocycler (Thermo Fisher Scientific, Waltham, MA USA). A 1.3 kb segment of the *pol* gene, covering all 99 *protease* (PR) codons and the first 300 codons of the *reverse transcriptase* (RT) gene, was amplified using proprietary manufacturer-supplied primers included within the Applied Biosystems HIV genotyping workflow and high-fidelity Platinum Taq DNA polymerase (Thermo Fisher Scientific, Waltham, MA, USA). Amplicons were visualised on 1% agarose gels, purified, and sequenced bidirectionally on a SeqStudio Genetic Analyzer (Thermo Fisher Scientific, Waltham, MA USA), using standard Sanger sequencing. *Integrase* sequencing was not performed.

### Sequence assembly and bioinformatics analysis

Raw reads were trimmed to a quality threshold of 30 (Q > 30) and assembled into consensus sequences using Geneious Prime (version 2024.0.7; Biomatters Ltd, Auckland, New Zealand) [21]. Discordant base calls identified during assembly were resolved through manual inspection. DRMs and viral subtypes were determined using the Stanford University HIV Drug Resistance Database (HIVdb) algorithm (version 9.8; accessed August 2025) [22]. Consensus sequences were aligned to the HXB2 reference (K03455) and manually inspected in AliView (version 1.27) [23]. Quality-control procedures included phylogenetic screening for potential sequence contamination or clustering anomalies, hypermutation using Hypermut 2.0 [24], and recombination using the Recombination Identification Program (RIP) (version 3.0) [25] prior to phylogenetic analysis. Major DRM codons were removed before maximum-likelihood phylogenetic inference in IQ-TREE (version 2.2.0) [26] under the general time reversible model with invariant sites and gamma-distributed rate variation (GTR + I+G) substitution model with 1,000 ultrafast bootstrap replicates. Reference subtype sequences (A, B, C, D) were obtained from the Los Alamos HIV Sequence Database, and trees were visualised in FigTree (version 1.4.4) [27]. Phylogenetic analysis was performed primarily for sequence quality assessment and subtype context. All consensus sequences were deposited in NCBI GenBank in October 2025, under accession numbers PX475350 to PX475646.

### Statistical analysis

All analyses were performed in Stata (version 17) (StataCorp, College Station, TX, USA) [28]. Continuous variables were summarised using medians and interquartile ranges (IQR), while categorical variables were presented as frequencies and percentages. Associations were explored using Fisher’s exact, chi-square, or Mann–Whitney U tests as appropriate. Multivariable logistic regression identified factors independently associated with major PI DRMs. Variables were selected a priori based on clinical relevance using a theory-driven approach. Adjusted odds ratios (aORs) with 95% confidence intervals (CIs) were reported. Model fit was assessed using the Hosmer–Lemeshow goodness-of-fit test. Multicollinearity among covariates was assessed using variance inflation factors (VIF), with a threshold of > 10 for severe collinearity. Where collinearity was identified, correlated covariates were assessed separately, and the most parsimonious biologically plausible model was retained. Missing data were handled using complete-case analysis. Statistical significance was defined as a two-sided p-value < 0.05.

### Study variables and definitions

The primary outcome was the presence of at least one major PI DRM, defined as any mutation classified as “major” by the Stanford University HIVdb algorithm. Covariates were derived from clinical records accompanying laboratory request forms and from laboratory-generated data. Continuous variables included age (years); duration since ART initiation (years); duration on PI-based second-line ART (years); and VL (copies/mL), measured at the time of VF. VL values were log₁₀-transformed for regression analyses. Categorical variables included sex (male or female), and current PI-based ART regimen. Province of referral was summarised descriptively only. Age was analysed continuously in regression models to avoid arbitrary categorisation and categorically for descriptive analyses only. Descriptive age categories were aligned with programmatic groupings used in national HIV treatment guidelines. The presence of reverse transcriptase inhibitor (RTI) resistance mutations, encompassing both nucleoside (NRTI) and non-nucleoside (NNRTI) mutations, was determined using the Stanford University HIVdb algorithm and summarised descriptively to characterise patterns of multiclass HIVDR. Thymidine analogue mutations (TAMs) were defined as the presence of zidovudine (AZT)- or stavudine(d4T)-associated resistance pathways, including M41L, D67N/E, K70R, L210W, T215F/Y, and K219Q/E, based on the Stanford University HIVdb classification system.

### Secondary outcomes

Secondary analyses included estimation of the prevalence of single-class, dual-class, and triple-class resistance. Single-class resistance was defined as the presence of DRMs within one ART drug class (major PI, NRTI, or NNRTI), dual-class resistance as the presence of DRMs within any two classes, and triple-class resistance as the co-occurrence of major PI-, NRTI-, and NNRTI-associated DRMs. Predicted antiretroviral (ARV) drug susceptibility profiles were evaluated using the Stanford University HIVdb scoring system, and categorised as susceptible (S), potential low-level resistance (PLLR), low-level resistance (LLR), intermediate resistance (I), or high-level resistance (HLR) based on cumulative mutation penalty scores.

### Bias minimisation

Measures were implemented to minimise potential sources of bias during sample processing and analysis. Standardised laboratory protocols, centralised processing at a single reference laboratory (BRTI), and standardised Stanford University HIVdb interpretation were used to reduce information bias and ensure consistency in sequence assembly and mutation calling. Treatment history and ART regimens were cross verified using clinical and laboratory records. Sequence quality-control procedures included phylogenetic, hypermutation, and recombination screening prior to analysis. Multivariable analyses adjusted for clinically relevant demographic and clinical covariates selected a priori.

### Ethical approval

Ethical approval was obtained from the BRTI Institutional Review Board (API78/2022) and the Medical Research Council of Zimbabwe (MRCZ) (A/2954). Permission to conduct the study was obtained from the Ministry of Health and Child Care (MoHCC). Retrospective analysis of archived specimens and de-identified clinical data was conducted under MRCZ waiver provisions. All data were anonymised using unique identifiers prior to analysis.

## Results

### Study population

**Fig. 1:**
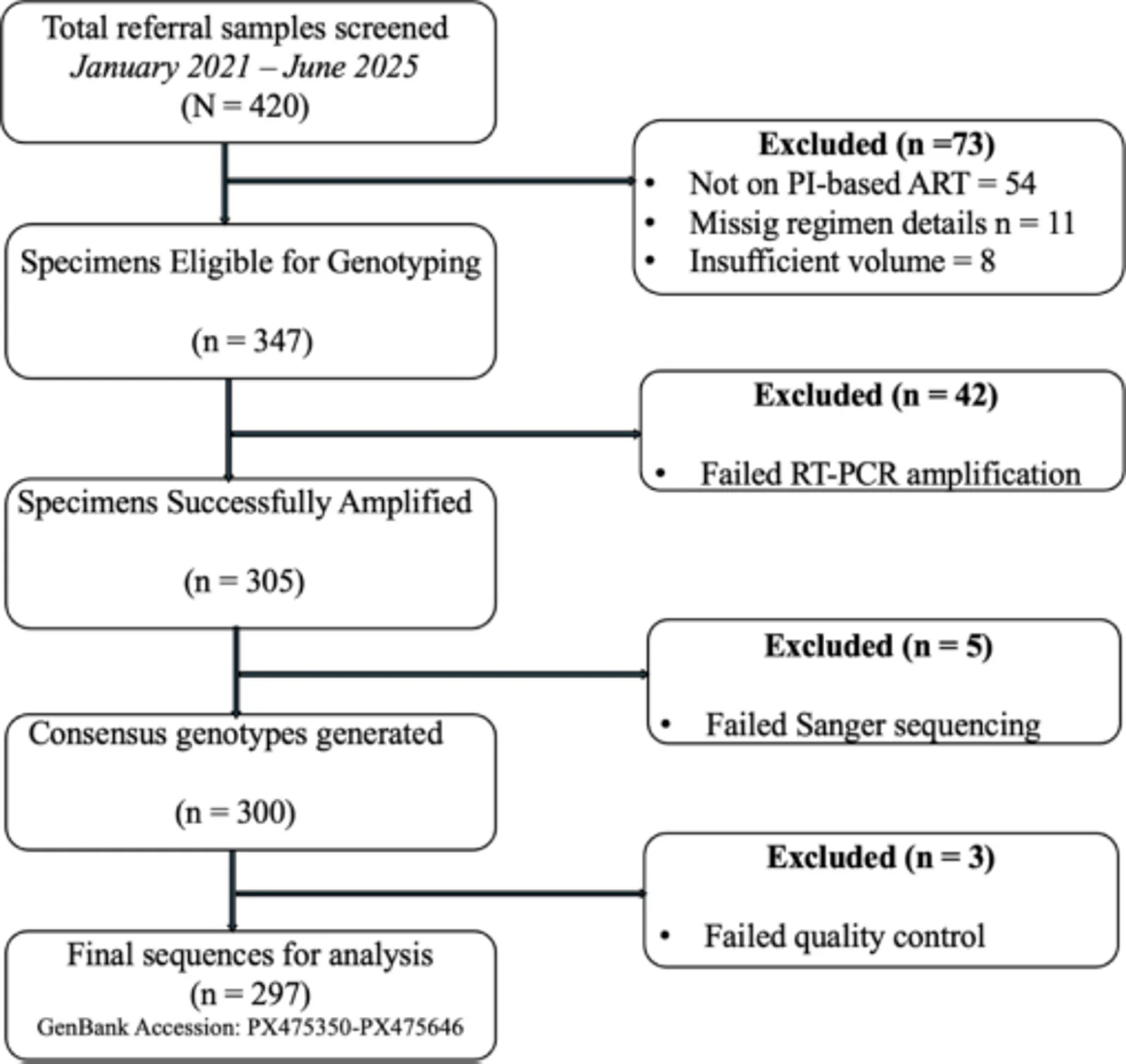
Flow diagram of specimen screening, genotyping, sequencing and quality-control procedures for inclusion in the final HIV drug resistance analysis. ART, antiretroviral therapy; PI, protease inhibitor; RT-PCR, real-time polymerase chain reaction. Eligibility was restricted to participants receiving PI-based second-line ART with virological failure (viral load ≥ 1,000 copies/mL). Sequences failing amplification, sequencing, or quality-control procedures were excluded from the final analysis

A total of 420 HIVDR referral specimens were screened for eligibility. Of these, 73 (17.4%) were excluded because participants were not receiving PI-based second-line ART (*n* = 54), ART regimen information was missing (*n* = 11), or specimen volume was insufficient (*n* = 8). The remaining 347 eligible specimens underwent genotyping. Amplification failed in 42 of 347 specimens (12.1%), and sequencing failed or yielded poor-quality sequences in five specimens (1.4%), resulting in 300 successfully generated sequences. Following sequence quality control, three sequences (1.0%) were excluded because of suspected contamination, leaving 297 sequences in the final analysis (Fig. 1). HIV-1 subtype C predominated among study participants, with one participant infected with subtype B.

### Participant characteristics

Baseline characteristics of the study population stratified by the presence or absence of major PI DRMs are presented in Table 1. The median age was 36 years (IQR 22–48), and 149 participants (50.2%) were male. Participants aged < 20 years, comprising paediatric and adolescent individuals, represented 14.5% (43/297) of the study population, while the largest age group was 20–39 years (41.1%, 122/297). Participants had been on PI-based second-line ART for a median of 4 years (IQR 3–7). The most frequently prescribed second-line ART regimen was abacavir + lamivudine + ritonavir-boosted atazanavir (ABC + 3TC + ATV/r), used by 131/297 participants (44.1%). Participants were referred from all 10 provinces of Zimbabwe, reflecting the national coverage of the Zimbabwe HIVDR Programme. Harare (38.7%) and Bulawayo (30.6%) contributed the largest proportions of specimens. Compared with participants without major PI DRMs, those with major PI DRMs were older (median 42 vs. 27 years, *P* < 0.001), more likely to be male (56.2%, *P* = 0.042), and had similar VLs (median 4.7 vs. 4.6 log₁₀ copies/mL, *P* = 0.17). RT-associated DRMs were significantly more frequent among participants with major PI DRMs, with NRTI-associated DRMs detected in 98.0% versus 41.0% (*P* < 0.001) and NNRTI-associated DRMs in 93.5% versus 64.6% (*P* < 0.001).

**Table 1:** Baseline participant characteristics stratified by the presence or absence of major PI DRMs

Variable	All participants (*n* = 297)	Without major PI DRMs (*n* = 144)	With major PI DRMs (*n* = 153)	*P*-value
**Sex**				**0.042**
Male, n (%)	149 (50.2%)	63 (43.8%)	86 (56.2%)	
**Age**,** years**				**< 0.001**
Median (IQR)	36 (22–48)	27 (20–42)	42 (27–50)	
Age categories, n (%)*				
< 10 years	7 (2.4%)	3 (2.1%)	4 (2.6%)	
10–19 years	36 (12.1%)	24 (16.7%)	12 (7.8%)	
20–39 years	122 (41.1%)	70 (48.6%)	52 (34.0%)	
40–59 years	111 (37.4%)	38 (26.4%)	73 (47.7%)	
≥ 60 years	14 (4.7%)	6 (4.2%)	8 (5.2%)	
**Viral load**,** log₁₀ copies/mL**				0.17
Median (IQR)	4.6 (4.0–5.2)	4.6 (4.0–5.1)	4.7 (4.0–5.3)	
**NRTI mutations**,** n (%)**	209 (70.4%)	59 (41.0%)	150 (98.0%)	**< 0.001**
**NNRTI mutations**,** n (%)**	236 (79.5%)	93 (64.6%)	143 (93.5%)	**< 0.001**
**Time since ART initiation**,** years**^**†**^				0.54
Median (IQR)	13 (9–15)	13 (9–15)	13 (10–15)	
**Duration on PI-based ART**,** years**^**‡**^				0.29
Median (IQR)	4 (3–7)	4 (3–6)	5 (3–7)	
**Second-line ART regimen**,** n (%)**				0.20
ABC + 3TC + ATV/r	131 (44.1%)	66 (45.8%)	65 (42.5%)	
AZT + 3TC + ATV/r	76 (25.6%)	33 (22.9%)	43 (28.1%)	
TDF + 3TC + ATV/r	65 (21.9%)	34 (23.6%)	31 (20.3%)	
ABC + 3TC + LPV/r	19 (6.4%)	10 (6.9%)	9 (5.9%)	
AZT + 3TC + LPV/r	5 (1.7%)	0 (0.0%)	5 (3.3%)	
TDF + 3TC + DRV/r	1 (0.3%)	1 (0.7%)	0 (0.0%)	

3TC, lamivudine; ABC, abacavir; ART, antiretroviral therapy; ATV/r, ritonavir-boosted atazanavir; AZT, zidovudine; DRMs, drug resistance mutations; IQR, interquartile range; LPV/r, ritonavir-boosted lopinavir; NNRTI, non-nucleoside reverse transcriptase inhibitor; NRTI, nucleoside reverse transcriptase inhibitor; PI, protease inhibitor; TDF, tenofovir disoproxil fumarate. Age categories are presented to describe the demographic composition of the study population. Statistical comparisons involving age were performed using the continuous age variable (median [IQR]); therefore, no p-values are presented for the age categories

^*^ Seven participants had missing age data and were excluded from age category summaries

^†^Time since ART initiation data were missing for 30 participants

^‡^ Duration on PI-based ART data were missing for 14 participants

### Prevalence and patterns of resistance

**Fig. 2:**
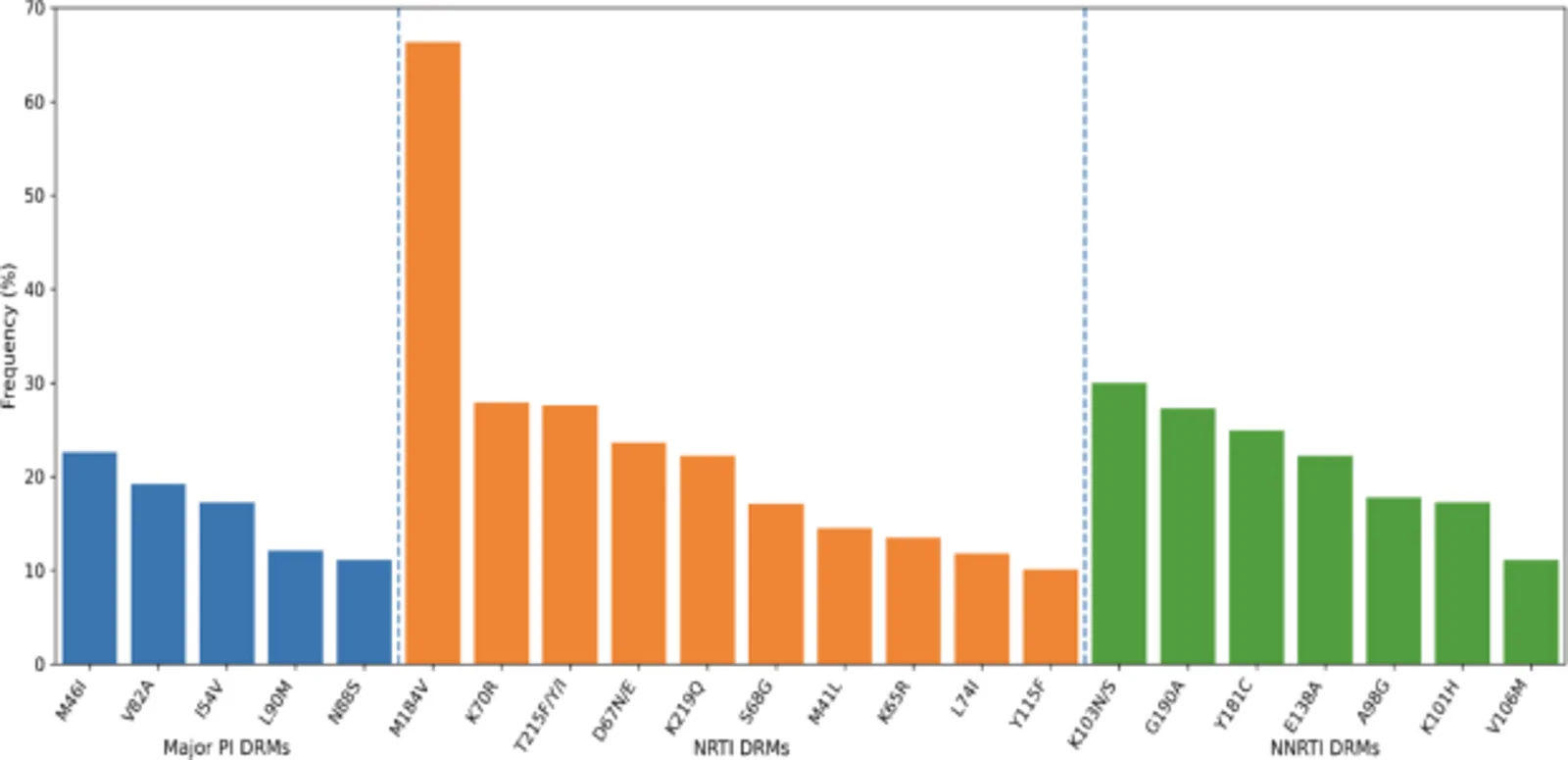
Frequency of HIV drug resistance mutations among participants with virological failure on PI-based second-line ART. DRMs, Drug resistance mutations. Bars represent the proportion of participants with each mutation. Mutations are grouped according to antiretroviral drug class: major protease inhibitor (PI) drug resistance mutations (blue), nucleoside reverse transcriptase inhibitor (NRTI) mutations (orange), and non-nucleoside reverse transcriptase inhibitor (NNRTI) mutations (green). Only mutations with prevalence ≥ 10% are displayed for visual clarity; the complete mutation profile is provided in Supplementary Table 1

Overall, 153 of 297 participants (51.5%, 95% CI 45.8–57.2%) had at least one major PI DRM. The median number of major PI DRMs per participant was 2 (IQR 1–3). The most frequently observed major PI DRMs were M46I (22.6%), V82A (19.2%), and I54V (17.2%) (Fig. 2; Supplementary Table 1). Among participants with major PI DRMs, accessory PI-associated mutations were detected in 137/153 (89.5%), with L89T (20.9%), Q58E (16.5%), and K20T (15.2%) being the most common. Any PI-associated mutation was detected in 172/297 participants (57.9%).

Evaluation of mutually exclusive resistance patterns showed that 85.5% (254/297) of participants had at least one DRM across the three major ART drug classes. Single-class resistance was observed in 22.6% (67/297) of participants and was predominantly driven by NNRTI-associated DRMs only (19.2%, 57/297), followed by NRTI-only (2.7%, 8/297) and major PI-only profiles (0.7%, 2/297). RT-associated DRMs were also frequent, with NNRTI-associated DRMs detected in 236/297 participants (79.5%) and NRTI-associated DRMs in 209/297 (70.4%). The most common NNRTI- and NRTI-associated DRMs were K103N/S (30.0%) and M184V (66.3%), respectively. TAMs were also frequently observed, particularly K70R (27.9%), T215F/Y/I (27.6%), D67N/E (23.6%), and K219Q (22.2%) (Fig. 2). Dual-class resistance patterns were present in 15.1% (45/297) of participants, with the NRTI + NNRTI combination being the most frequent (12.1%, 36/297). Triple-class resistance was observed in 142/297 participants (47.8%, 95% CI 42.0–53.6%). Detailed class-specific prevalences are shown in Table 2 and Supplementary Table 2.

**Table 2:** Prevalence of overall and mutually exclusive HIV drug resistance profiles among participants with virological failure on PI-based second-line ART (*N* = 297)

Resistance Category	Frequency (*n*)	Percentage (%)	95% Confidence Interval (%)
Overall DRM Prevalence (Non-exclusive)			
No DRMs Detected	43	14.5	10.7–19.0
Major PI DRMs ^†^	153	51.5	45.8–57.2
Any PI mutation (major or accessory) ^‡^	172	57.9	52.2–63.5
NRTI DRMs	209	70.4	64.9–75.5
NNRTI DRMs	236	79.5	74.5–83.9
Mutually Exclusive Resistance Profiles^§^			
Single-class resistance			
NNRTI only	57	19.2	14.9–24.2
NRTI only	8	2.7	1.2–5.2
Major PI only	2	0.7	0.1–2.4
Dual-class resistance			
NRTI + NNRTI	36	12.1	8.7–16.4
Major PI + NRTI	8	2.7	1.2–5.2
Major PI + NNRTI	1	0.3	0.0–1.9
Triple-class resistance ^¶^			
Major PI + NRTI + NNRTI	142	47.8	42.0–53.6
Total with ≥ 1 DRM detected	254	85.5	81.0–89.3

DRMs, drug resistance mutations; PI, protease inhibitor; NRTI, nucleoside reverse transcriptase inhibitor; NNRTI, non-nucleoside reverse transcriptase inhibitor; CI, confidence interval; ART, antiretroviral therapy. This table presents descriptive prevalence estimates only. Percentages and 95% confidence intervals are reported for each resistance category; no statistical comparisons between resistance categories were performed

† Major PI DRMs were defined according to the Stanford University HIV Drug Resistance Database (version 9.8) as the presence of at least one major protease resistance mutation

‡ Any PI mutation was defined as the presence of at least one major or accessory protease-associated mutation

§ Single-, dual-, and triple-class resistance categories were mutually exclusive and defined according to the presence or absence of major PI-, NRTI-, and NNRTI-associated DRMs

¶ Triple-class resistance was defined as the co-occurrence of at least one major PI DRM, one NRTI DRM, and one NNRTI DRM within a single sequence

### Predicted antiretroviral drug susceptibility

**Fig. 3:**
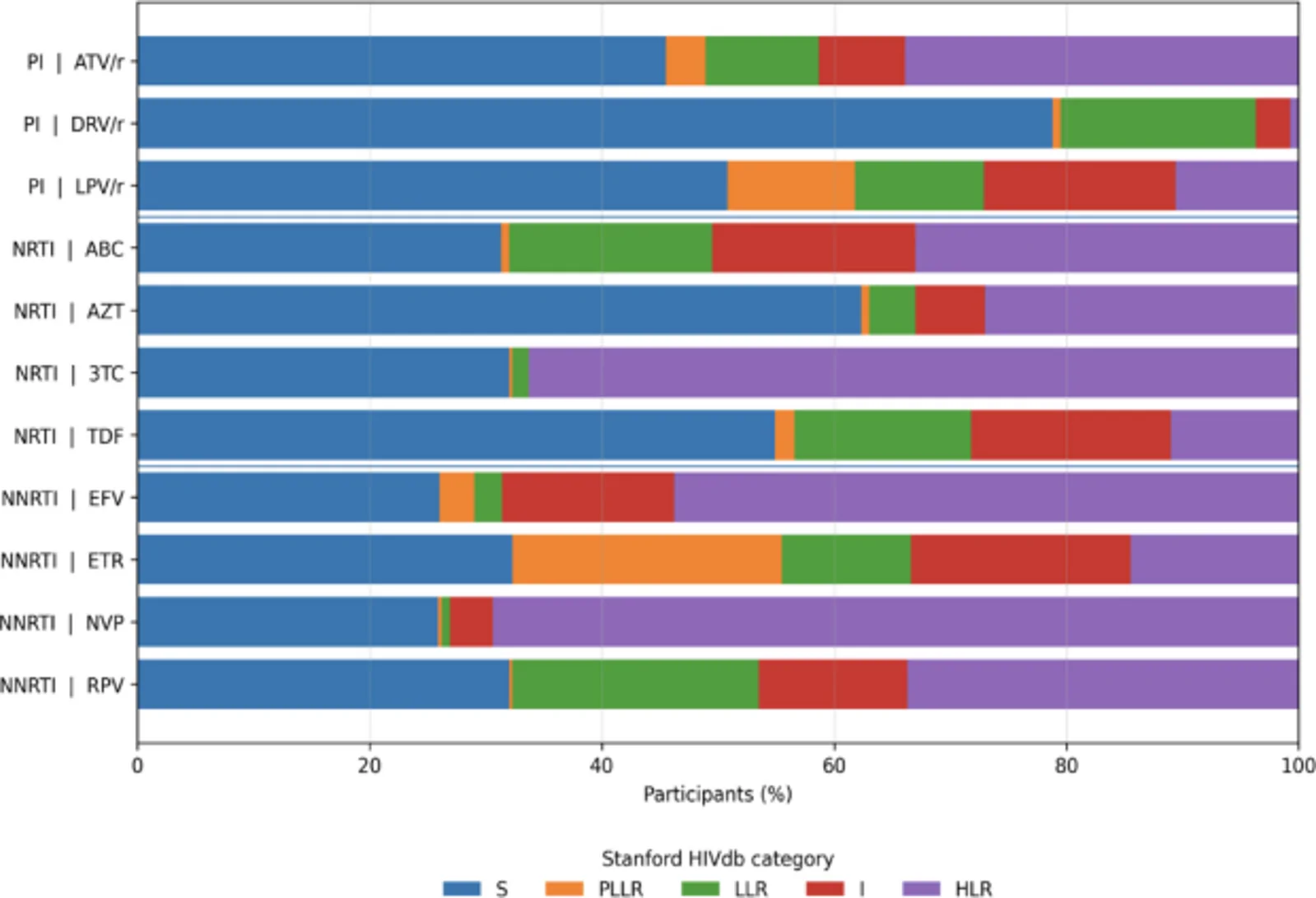
Predicted antiretroviral drug susceptibility profiles among participants with virological failure on PI-based second-line ART. Protease inhibitors (PIs) included atazanavir/ritonavir (ATV/r), darunavir/ritonavir (DRV/r), and lopinavir/ritonavir (LPV/r). Nucleoside reverse transcriptase inhibitors (NRTIs) included abacavir (ABC), zidovudine (AZT), lamivudine (3TC), and tenofovir disoproxil fumarate (TDF). Non-nucleoside reverse transcriptase inhibitors (NNRTIs) included efavirenz (EFV), etravirine (ETR), nevirapine (NVP), and rilpivirine (RPV). Susceptibility categories were assigned using the Stanford HIV Drug Resistance Database scoring system: S, susceptible; PLLR, potential low-level resistance; LLR, low-level resistance; I, intermediate resistance; and HLR, high-level resistance. Each bar represents the proportional distribution of predicted susceptibility categories for each antiretroviral drug

Predicted antiretroviral (ARV) drug susceptibility profiles are summarised in Fig. 3 and Supplementary Table 3. Among PIs, HLR to DRV/r was rare (< 1%), and overall predicted susceptibility to DRV/r remained high, with 78.8% of participants classified as susceptible. In contrast, reduced susceptibility was observed for atazanavir/ritonavir (ATV/r) and lopinavir/ritonavir (LPV/r), with only 45.5% and 50.8% of participants classified as susceptible, respectively. HLR was most pronounced for ATV/r (34.0%), compared with 11.0% for LPV/r.

Among NRTIs, AZT and tenofovir (TDF) retained moderate levels of activity, with 62.3% and 54.9% of participants classified as susceptible, respectively. In contrast, lamivudine (3TC) demonstrated high levels of resistance, with 66.3% of participants classified as having HLR. Abacavir (ABC) also showed high resistance, with 33.0% of participants exhibiting HLR.

Predicted susceptibility to NNRTIs was generally low. Only 26.0% and 25.9% of participants were classified as susceptible to efavirenz (EFV) and nevirapine (NVP), respectively, while HLR was observed in 53.9% and 69.4%. Etravirine (ETR) and rilpivirine (RPV) demonstrated intermediate susceptibility profiles, with susceptibility observed in 32.3% and 32.0% of participants, respectively.

### Factors associated with major PI drug resistance mutations

In multivariable logistic regression (Table 3), increasing age (aOR 1.04 per year increase, 95% CI 1.02–1.06; *P* < 0.001), higher VL (aOR 1.55, 95% CI 1.10–2.18; *P* = 0.011), and male sex (aOR 1.59, 95% CI 1.03–2.49; *P* = 0.044) were independently associated with higher odds of major PI DRMs. In contrast, time on ART and duration of exposure to PI-based regimens were not independently associated with major PI DRMs, *P* = 0.692 and *P* = 0.453 respectively.

**Table 3:** Multivariable logistic analysis of factors associated with major protease inhibitor drug resistance mutations (complete-case analysis; *n* = 247)

Variable	aOR	95% CI	*P*-value
Age (per year increase) *	1.04	1.02–1.06	**< 0.001**
**Male sex**	1.59	1.03–2.49	**0.044**
**Viral load (log₁₀ copies/mL)**	1.55	1.10–2.18	**0.011**
**Time on ART (years)** ^†^	1.03	0.95–1.11	0.453
**Duration on PI-based ART (years)** ^‡^	0.98	0.87–1.10	0.692

aOR, adjusted odds ratio; ART, antiretroviral therapy; CI, confidence interval; PI, protease inhibitor. Multivariable logistic regression was performed using complete-case analysis, participants with missing covariate data were excluded from the regression model

^*^Age data were missing for seven participants

^†^Time since ART initiation data were missing for 30 participants

^‡^ Duration on PI-based ART data were missing for 14 participants

## Discussion

This study provides contemporary evidence on the burden and patterns of HIVDR among PLWH experiencing VF on PI-based second-line ART in Zimbabwe during the DTG era. The observed prevalence of major PI DRMs (51.5%, 95% CI 45.8–57.2%) indicates frequent PI resistance among treatment-experienced individuals receiving second-line ART. The prevalence observed in this study is higher than that reported in several earlier Zimbabwean and regional studies [8, 9, 19], although comparisons should be interpreted cautiously because the study population comprised treatment-experienced individuals referred for GRT after confirmed VF, and because of differences in treatment duration, VL thresholds, sequencing methodologies, and resistance interpretation algorithms. Nevertheless, previous studies across SSA have similarly demonstrated progressive accumulation of PI-associated resistance among individuals experiencing prolonged second-line VF^6,7,10,11^. In many LMICs, interruptions in routine VL monitoring and limited access to real-time GRT may permit ongoing viraemia under partial drug pressure, facilitating continued resistance selection [12].

Nearly half of participants (47.8%) demonstrated triple-class resistance involving major PI-, NRTI- and NNRTI-associated DRMs, reflecting prolonged exposure to sequential ART regimens among individuals experiencing VF on second-line ART. Although boosted PIs possess a high genetic barrier to resistance, the near-universal presence of RT-associated DRMs among participants with major PI DRMs suggests prolonged VF under partially compromised regimens. The high prevalence of M184V (66.3%) and K103N/S (34.0%) is compatible with historical exposure to 3TC- and NNRTI-based first-line regimens widely used in Zimbabwe and across SSA [29, 30]. Frequent detection of TAMs, particularly K70R, T215F/Y/I, and D67N/E, further supports prolonged exposure to failing NRTI-containing regimens and cumulative resistance evolution within participants. In addition to conferring HLR to 3TC and FTC, M184V has also been associated with reduced viral fitness [31]. Although RT mutations do not mechanistically induce PI mutations, the loss of NRTI backbone support may reflect sustained viral replication under PI selective pressure, contributing to cumulative resistance accumulation during persistent virological failure [32].

The observed PI mutation spectrum, dominated by M46I (22.6%), V82A (19.2%), and I54V (17.2%), is consistent with established LPV/r and ATV/r resistance pathways [6, 33]. Although M46I was the most frequent major PI DRM, its isolated impact on PI susceptibility is generally limited and, in isolation, is often insufficient to warrant regimen change [34]. Resistance interpretation was therefore based on cumulative mutation profiles and Stanford University HIVdb-derived susceptibility scores. Similar considerations apply to accessory PI mutations, which may contribute to resistance primarily in combination with other major DRMs. Despite high levels of resistance to ATV/r and LPV/r, predicted susceptibility to DRV/r remained largely preserved (78.8%), with HLR observed in fewer than 1% of participants. This supports the continued role of DRV/r-based third-line regimens as durable salvage options for heavily treatment-experienced individuals in RLS as previously reported [6, 11]. Despite widespread DTG rollout, PI-based regimens remain important components of second- and subsequent-line ART, particularly among individuals with prior NNRTI failure or complex treatment histories. Continued surveillance of PI resistance therefore remains important for informing long-term ART sequencing during the transition toward INSTI-based regimens, especially as the long-term evolution of PI resistance patterns in the DTG era remains incompletely characterised [5].

Increasing age, higher VL, and male sex were independently associated with major PI DRMs. The association between major PI DRMs with increasing age likely reflects longer cumulative ART exposure and sequential transitions through multiple regimens, including extended exposure to NNRTI-based first-line ART prior to PI-based second-line therapy. Similar associations between treatment exposure and resistance accumulation have been reported among treatment-experienced populations failing second-line ART in SSA [6, 35, 36]. Although the study included paediatric, adolescent, and adult participants, individuals aged < 20 years represented only 14.5% of the study population, limiting statistical power for robust age-stratified analyses. In addition, the Zimbabwe national treatment guidelines during the study period applied broadly similar PI-based second-line ART principles across age groups, with differences primarily related to weight-based dosing and formulation adaptations [16].

Higher VL was independently associated with major PI DRMs in the present study. Few studies have specifically evaluated predictors of major PI DRMs among individuals with confirmed second-line VF. Although a previous South African study evaluated VL as a potential predictor of major PI DRMs, the association was not retained in the final multivariable model [9]. The significant association observed in our study therefore provides additional evidence that the degree of viraemia may help identify individuals at increased risk of harbouring PI resistance. Biologically, persistent viraemia reflects ongoing viral replication under ARV drug pressure, facilitating the selection and accumulation of drug-resistant variants, including major PI DRMs. These findings reinforce the importance of routine VL monitoring within Zimbabwe’s HIV programme and support timely GRT among individuals with persistent VF despite adherence support, enabling earlier optimisation of ART in accordance with WHO recommendations [4]. Male sex has also been associated with poorer virological outcomes in some ART programmes in LMICs, potentially related to delayed clinical presentation, interruptions in care, alcohol use and suboptimal adherence and broader social and structural barriers affecting continuity of HIV care [5], [37–39], although these factors were not specifically assessed in the present study. Our findings nevertheless contrast with reports from some South African cohorts in which second-line VF and major PI DRMs were more frequently observed among women [40], suggesting possible regional heterogeneity in treatment experiences and programme dynamics.

Several strengths of this study should be highlighted. This study utilised a contemporary multi-provincial dataset to evaluate PI-associated HIVDR among individuals experiencing VF on second-line ART in Zimbabwe. By incorporating specimens collected through the National HIVDR Programme across multiple provinces, the study provides important insight into resistance patterns among treatment-experienced populations within routine public-sector ART programmes. In addition, standardised laboratory and bioinformatics quality-control procedures, including DRM-codon stripping, phylogenetic quality assessment, hypermutation screening and recombination analysis, ensured the quality and reliability of the sequence data.

Nevertheless, several limitations should also be acknowledged. The retrospective cross-sectional design limits causal and temporal inference regarding the emergence and evolution of specific DRMs. We cannot determine whether high VL preceded the development of DRMs or vice versa. Longitudinal studies are needed to clarify this temporal relationship. Additionally, limiting inclusion to successfully sequenced specimens introduces potential selection bias. However, post-hoc comparison of successfully and unsuccessfully genotyped specimens demonstrated no major differences in median age or VL, suggesting that amplification failures were more likely related to specimen degradation during long-term storage than systematic participant differences. Data on adherence, treatment interruptions, and prior resistance history were unavailable. Although specimens were obtained from multiple provinces, the study was not based on a nationally representative sampling framework, and findings may therefore not be fully generalisable to all PLWH receiving second-line ART in Zimbabwe. Because the study focused on individuals with established VF on second-line ART, the findings reflect acquired post-treatment resistance and cannot be extrapolated to transmitted HIVDR among ART-naïve populations. Finally, the use of conventional population (Sanger) sequencing may have underestimated low-frequency minority resistance variants present below approximately 15–20% of the viral population [41].

## Conclusions

In conclusion, this study demonstrates a high burden of major PI DRMs and multiclass HIVDR among PLWH experiencing VF on PI-based second-line ART in Zimbabwe. The widespread accumulation of resistance across multiple ART classes underscores the importance of strengthened HIVDR surveillance, timely VL monitoring, and expanded access to GRT within national HIV treatment programmes. Despite the high prevalence of resistance, predicted susceptibility to DRV/r remained largely preserved, supporting the continued role of DRV/r-based third-line regimens for treatment-experienced individuals. Continued HIVDR surveillance and expanded access to GRT remain important for sustaining long-term ART effectiveness in the DTG era.

## Supplementary Information

Supplementary Material 1

## Data Availability

The HIV-1 protease and reverse transcriptase sequences generated during the current study are available in the NCBI GenBank repository, accession numbers PX475350 to PX475646, https://www.ncbi.nlm.nih.gov/nuccore/?term=PX475350:PX475646[accn]. All other data generated or analysed during this study are included in this published article and its supplementary information files.

## Abbreviations

3TC: Lamivudine
ABC: Abacavir
ART: Antiretroviral therapy
ATV/r: Atazanavir/ritonavir
APOBEC: Apolipoprotein B mRNA editing catalytic polypeptide-like
BRTI: Biomedical Research and Training Institute
CI: Confidence interval
DNA: Deoxyribonucleic acid
DRM: Drug resistance mutation
DRV/r: Darunavir/ritonavir
DTG: Dolutegravir
FTC: Emtricitabine
GRT: Genotypic resistance testing
HIV: Human immunodeficiency virus
HIVDR: HIV drug resistance
INSTI: Integrase strand transfer inhibitor
LMICs: Low- and middle-income countries
LPV/r: Lopinavir/ritonavir
MoHCC: Ministry of Health and Child Care
NNRTI: Non-nucleoside reverse transcriptase inhibitor
NRTI: Nucleoside reverse transcriptase inhibitor
PI: Protease inhibitor
PLWH: People living with HIV
RNA: Ribonucleic acid
RT: Reverse transcriptase
RT-PCR: Reverse transcription polymerase chain reaction
SSA: Sub-Saharan Africa
TAMs: Thymidine analogue mutations
VF: Virological failure
VL: Viral load
WHO: World Health Organization
