# Supplementary material for: Major protease inhibitor drug resistance mutations among patients with virological failure on second-line antiretroviral therapy in Zimbabwe: a retrospective cross-sectional study
Source: Virol J. 2026 Jul 10;23:239. doi: 10.1186/s12985-026-03255-6 (PMC13647759; doi:10.1186/s12985-026-03255-6)
Supplement: Supplementary file 1 [file 12985_2026_3255_MOESM1_ESM.docx]

**Supplementary Tables**

**Major protease inhibitor drug resistance mutations among patients with virological failure on second-line antiretroviral therapy in Zimbabwe: a retrospective cross-sectional study**

**Supplementary Table 1:** Distribution of PI-, NRTI-, and NNRTI-associated drug resistance mutations among 297 HIV-1 sequences**.**

| Mutation Category | Mutation | Frequency (n) | Percentage (%) |
| --- | --- | --- | --- |
| Major PI-associated DRMs | | | |
|  | M46I | 67 | 22.6% |
|  | V82A | 57 | 19.2% |
|  | I54V | 53 | 17.2% |
|  | L90M | 36 | 12.1% |
|  | N88S | 33 | 11.1% |
|  | I50L | 27 | 9.1% |
|  | I84V | 21 | 7.1% |
|  | V32A | 19 | 6.4% |
|  | L76V | 6 | 2.0% |
|  | I47V | 4 | 1.3% |
| Accessory PI-associated DRMs | | | |
|  | L89T | 62 | 20.9% |
|  | Q58E | 49 | 16.5% |
|  | K20T | 45 | 15.2% |
|  | G73S | 33 | 11.1% |
|  | L23I | 29 | 9.8% |
|  | K43T | 28 | 9.4% |
|  | L33F | 18 | 6.1% |
|  | L10F | 14 | 4.7% |
|  | F53L/Y | 12 | 4.0% |
|  | T74P | 11 | 3.7% |
|  | L24I | 10 | 3.4% |
| TAM-associated NRTI DRMs | | | |
|  | K70R | 83 | 27.9% |
|  | T215F/Y/I | 82 | 27.6% |
|  | D67N/E | 70 | 23.6% |
|  | K219Q | 66 | 22.2% |
|  | M41L | 43 | 14.5% |
|  | L210W | 21 | 7.1% |
| **Non-TAM NRTI-associated DRMs** | | | |
|  | M184V | 197 | 66.3% |
|  | S68G | 53 | 17.1% |
|  | K65R | 40 | 13.5% |
|  | L74I | 35 | 11.8% |
|  | Y115F | 30 | 10.1% |
|  | V75M | 26 | 8.8% |
|  | A62V | 21 | 7.1% |
|  | T69D | 18 | 6.1% |
|  | E44D | 13 | 4.4% |
|  | F77L | 8 | 2.7% |
|  | Q151M | 3 | 1.0% |
| NNRTI-associated DRMs | | | |
|  | K103N/S | 89 | 30.0% |
|  | G190A | 81 | 27.3% |
|  | Y181C | 74 | 24.9% |
|  | E138A | 66 | 22.2% |
|  | A98G | 53 | 17.8% |
|  | K101H | 51 | 17.2% |
|  | V106M | 33 | 11.1% |
|  | V108I | 24 | 8.1% |
|  | H221Y | 21 | 7.1% |
|  | V179D | 17 | 5.7% |
|  | F227L | 13 | 4.4% |
|  | Y188L | 13 | 4.4% |
|  | L100I | 10 | 3.4% |
|  | M230L | 2 | 0.7% |
|  | G109A | 1 | 0.3% |

DRMs, drug resistance mutations; NNRTI, non-nucleoside reverse transcriptase inhibitor; NRTI, nucleoside reverse transcriptase inhibitor; PI, protease inhibitor; TAMs, thymidine analogue mutations. Mutation classification was based on the Stanford HIV Drug Resistance Database (HIVdb version 9.8).

**Supplementary Table 2**: Mutually exclusive drug-class resistance patterns according to baseline PI-based second-line ART regimen.

| Resistance Pattern | All participants (n = 297) ^‡^ | ABC/3TC/ATV/r (n = 131) ^‡^ | AZT/3TC/ATV/r (n = 76) ^‡^ | TDF/3TC/ATV/r (n = 65) ^‡^ | ABC/3TC/LPV/r (n = 19) ^‡^ | AZT/3TC/LPV/r (n = 5) ^‡^ | TDF/3TC/LPV/r (n = 1) ^‡^ |
| --- | --- | --- | --- | --- | --- | --- | --- |
| No DRMs Detected | 43 (14.5%) | 16 (12.2%) | 10 (13.2%) | 13 (20.0%) | 4 (21.1%) | 0 (0.0%) | 0 (0.0%) |
| Single-class resistance^†^ | | | | | | | |
| Major PI DRMs only | 2 (0.7%) | 1 (0.8%) | 0 (0.0%) | 1 (1.5%) | 0 (0.0%) | 0 (0.0%) | 0 (0.0%) |
| NRTI DRMs only | 8 (2.7%) | 2 (1.5%) | 3 (3.9%) | 3 (4.6%) | 0 (0.0%) | 0 (0.0%) | 0 (0.0%) |
| NNRTI DRMs only | 57 (19.2%) | 32 (24.4%) | 10 (13.2%) | 10 (15.4%) | 4 (21.1%) | 0 (0.0%) | 1 (100.0%) |
| Dual-class resistance^†^ | | | | | | | |
| Major PI + NNRTI DRMs | 1 (0.3%) | 0 (0.0%) | 0 (0.0%) | 1 (1.5%) | 0 (0.0%) | 0 (0.0%) | 0 (0.0%) |
| Major PI + NRTI DRMs | 8 (2.7%) | 2 (1.5%) | 1 (1.3%) | 4 (6.2%) | 0 (0.0%) | 1 (20.0%) | 0 (0.0%) |
| NRTI + NNRTI DRMs | 36 (12.1%) | 15 (11.5%) | 11 (14.5%) | 6 (9.2%) | 4 (21.1%) | 0 (0.0%) | 0 (0.0%) |
| Triple-class resistance^†^ | | | | | | | |
| Major PI + NRTI + NNRTI | 142 (47.8%) | 63 (48.1%) | 41 (53.9%) | 27 (41.5%) | 7 (36.8%) | 4 (80.0%) | 0 (0.0%) |
| Total with ≥ 1 Mutation | 254 (85.5%) | 115 (87.8%) | 66 (86.8%) | 52 (80.0%) | 15 (78.9%) | 5 (100.0%) | 1 (100.0%) |

3TC, lamivudine; ABC, abacavir; ART, antiretroviral therapy; ATV/r, atazanavir/ritonavir; AZT, zidovudine; DRMs, drug resistance mutations; LPV/r, lopinavir/ritonavir; NNRTI, non-nucleoside reverse transcriptase inhibitor; NRTI, nucleoside reverse transcriptase inhibitor; PI, major protease inhibitor-associated drug resistance mutations; TDF, tenofovir disoproxil fumarate; VF, virological failure.

† Resistance patterns were mutually exclusive and defined according to the concurrent presence or absence of major PI-, NRTI-, and NNRTI-associated DRMs interpreted using the Stanford HIV Drug Resistance Database (HIVdb version 9.8)

‡ Percentages were calculated using the column denominator for each baseline ART regimen category. Percentages for regimen categories with small sample sizes should be interpreted cautiously.

**Supplementary Table 3*:*** Predicted antiretroviral drug susceptibility profiles derived from Stanford HIVdb interpretation among participants with VF on PI-based second-line ART.

|  | ****Drug Susceptibility Interpretation**** | | | | |
| --- | --- | --- | --- | --- | --- |
| ****ARV Drug**** | **S, n (%)** | **PLLR, n (%)** | **LLR, n (%)** | **I, n (%)** | **HLR, n (%)** |
| ****PIs**** | | | | | |
| ATV/r | 135 (45.5%) | 10 (3.4%) | 29 (9.8%) | 22 (7.4%) | 101 (34.0%) |
| DRV/r | 234 (78.8%) | 2 (0.7%) | 50 (16.8%) | 9 (3.0%) | 2 (0.7%) |
| LPV/r | 151 (50.8%) | 32 (11.0%) | 33 (11.1%) | 49 (16.5%) | 32 (11.0%) |
| ****NRTIs**** | | | | | |
| ABC | 93 (31.3%) | 2 (0.7%) | 52 (17.5%) | 52 (17.5%) | 98 (33.0%) |
| AZT | 185 (62.3%) | 2 (0.7%) | 12 (4.0%) | 18 (6.0%) | 80 (26.9%) |
| 3TC | 95 (32.0%) | 1 (0.3%) | 4 (1.4%) | 0 (0.0%) | 197 (66.3%) |
| TDF | 163 (54.9%) | 5 (1.7%) | 45 (15.2%) | 51 (17.2%) | 33 (11.1%) |
| ****NNRTIs**** | | | | | |
| EFV | 77 (26.0%) | 9 (3.0%) | 7 (2.4%) | 44 (14.8%) | 160 (53.9%) |
| ETR | 96 (32.3%) | 69 (23.2%) | 33 (11.1%) | 56 (18.9%) | 43 (14.5%) |
| NVP | 77 (25.9%) | 1 (0.3%) | 2 (0.7%) | 11 (3.7%) | 206 (69.4%) |
| RPV | 95 (32.0%) | 1 (0.3%) | 63 (21.2%) | 38 (12.8%) | 100 (33.7%) |

Susceptibility interpretations were derived using the Stanford HIV Drug Resistance Database (HIVdb version 9.8). Percentages were calculated using the total study population (n = 297) as the denominator. 3TC, lamivudine; ABC, abacavir; ARV, antiretroviral; ATV/r, atazanavir/ritonavir; AZT, zidovudine; DRV/r, darunavir/ritonavir; EFV, efavirenz; ETR, etravirine; HLR, high-level resistance; I, intermediate resistance; LLR, low-level resistance; LPV/r, lopinavir/ritonavir; NNRTI, non-nucleoside reverse transcriptase inhibitor; NRTI, nucleoside reverse transcriptase inhibitor; NVP, nevirapine; PI, protease inhibitor; PLLR, potential low-level resistance; RPV, rilpivirine; S, susceptible; TDF, tenofovir disoproxil fumarate.
